# The Effect of Multidisciplinary Lifestyle Intervention on the Pre- and Postprandial Plasma Gut Peptide Concentrations in Children with Obesity

**DOI:** 10.5402/2011/353756

**Published:** 2011-06-01

**Authors:** Rimke C. Vos, Hanno Pijl, Jan M. Wit, Erik W. van Zwet, Chris van der Bent, Euphemia C. A. M. Houdijk

**Affiliations:** ^1^Department of Pediatrics, Juliana Children's Hospital/Haga Hospital, Sportlaan 600, 2566 MJ the Hague, The Netherlands; ^2^Department of Endocrinology and Metabolism, Leiden University Medical Center, P.O. Box 9600, 2300 RC Leiden, The Netherlands; ^3^Department of Pediatrics, Leiden University Medical Center, P.O. Box 9600, 2300 RC Leiden, The Netherlands; ^4^Department of Medical Statistics, Leiden University Medical Center, P.O. Box 9600, 2300 RC Leiden, The Netherlands

## Abstract

*Objective*. This study aims to evaluate the effect of a multidisciplinary treatment of obesity on plasma concentrations of several gut hormones in fasting condition and in response to a mixed meal in children. *Methods*. Complete data were available from 36 obese children (age 13.3 ± 2.0 yr). At baseline and after the 3-month multidisciplinary treatment, fasting and postprandial blood samples were taken for glucose, insulin, ghrelin, peptide YY (PYY), and glucagon-like peptide 1 (GLP-1). *Results*. BMI-SDS was significantly reduced by multidisciplinary treatment (from 4.2 ± 0.7 to 4.0 ± 0.9, *P* < .01). The intervention significantly increased the area under the curve (AUC) of ghrelin (from 92.3 ± 18.3 to 97.9 ± 18.2 pg/L, *P* < .01), but no significant changes were found for PYY or GLP-1 concentrations (in fasting or postprandial condition). The insulin resistance index (HOMA-IR) remained unchanged as well. *Conclusion*. Intensive multidisciplinary treatment induced moderate weight loss and increased ghrelin secretion, but serum PYY and GLP-1 concentrations and insulin sensitivity remained unchanged.

## 1. Introduction

Childhood obesity has become a global problem [1]. Understanding the mechanisms that control energy balance and fuel flux in children is of paramount importance for the design of effective strategies to combat this health hazard. The gastrointestinal tract plays a critical role in the control of feeding behavior and fuel metabolism. In response to food intake, the gut produces a variety of hormones that inhibit food intake, promote glucose induced insulin release, and/or facilitate insulin action (e.g., glucagon-like peptide 1 (GLP-1), peptide YY (PYY)) [2–4]. Conversely, in the absence of food, the stomach produces ghrelin, which stimulates appetite to initiate feeding [4, 5]. 

In a number of studies, plasma levels of ghrelin in obese children were compared with those of normal weight controls. Generally, the plasma ghrelin concentration of obese children is lower in fasting condition [6–10], whereas the decline in response to glucose ingestion varies as a function of insulin sensitivity; the decline appears to be less profound in insulin resistant obese children [6, 11], although the data are equivocal [12]. Weight loss tends to normalize circulating ghrelin levels [8, 10, 13], although this is still a subject for debate [5, 14]. 

In fasting condition, plasma GLP-1 and PYY levels were reported to be similar in obese and lean children [9, 15–18], but in other studies lower PYY [19] and lower GLP-1 were found in obese children [20]. Postprandial concentrations of the two hormones are consistently lower in obese versus normal weight peers [15, 20, 21]. The few studies that have examined the effect of weight loss on the release of these hormones in obese children found significantly increased fasting PYY [19] and (surprisingly) decreased fasting GLP-1 [18] after weight reduction. 

Because gut hormones are intimately involved in the control of energy balance, better understanding of intestinal endocrine (mal) function in obese children may contribute to the development of strategies to treat their condition. Here we aim to evaluate the effect of a 3-month multidisciplinary intervention program to reduce the bodyweight of obese children on plasma gut hormone concentrations in fasting condition and in response to a standard meal.

## 2. Materials and Methods

### 2.1. Participants, Study Design, and Settings

This study is part of a randomized clinical trial on the effect of multidisciplinary treatment on childhood obesity [22]. Here the effects of lifestyle intervention on various clinical and physiological features of the obese children in the intervention group are analyzed. Characteristics of the obese control group as well as treatment effect over the course of 1 and 2 years followup were the topic of a previous study [23]. The study inclusion criteria were simple obesity (as defined by Cole [24]), age 8–17 years, and referral to a pediatrician. Potential participants were excluded if their knowledge of the Dutch language, intelligence, or social skills were insufficient to participate in the trial. Other exclusion criteria were use of medication that might have an effect on weight loss, medical comorbidity (e.g., hypothyroidism, high dose of glucocorticoids, diabetes mellitus) that could affect trial outcome, or previous enrollment in another cognitive behavioral treatment program with a focus on reducing body weight. Forty children were randomized to the intervention group, of whom 36 completed the treatment. The study was conducted according to the “Declaration of Helsinki”, and approval was obtained from the regional medical ethical committee South West Holland. All parents and children gave their written informed consent after they had been given detailed written explanations of the aims of the study, discomfort, and inconvenience, and the option to withdraw at any time.

### 2.2. Intervention

The multidisciplinary lifestyle treatment of the intervention group consisted of a screening phase of individual counseling of the children with their parents, followed by an intensive phase of group sessions during three months. The group treatment consisted of 7 group meetings for the children, 5 separate parent meetings, and 1 parent meeting together with the children. Meetings of 2.5 hours were held once every 2 weeks. The children meetings were held on weekday afternoons at the hospital. The 5 separate parent meetings were provided on weekdays after working hours. Subsequently, refresh follow-up sessions (2-3 sessions/year) were offered for a total period of two years (Table 1).

### 2.3. Clinical Measures

In this paper, the measurements taken at baseline (T1) and after 3 months treatment (T2) are analyzed and discussed. Weight was measured to the nearest of 0.1 kg using an electronic scale (SECA 911, Vogel & Halke, Hamburg, Germany) and height to the nearest of 0.1 cm with a stadiometer (Holtain, limited, Crymych, Dyfed, Britain) in underwear and barefoot by an experienced assistant. The BMI was calculated as weight/height squared (kg/m^2^). Subjects were classified as obese using BMI gender- and age-specific international cutoff levels [24]. BMI was expressed as standard deviation score (SDS) for Dutch references using the LMS method [26]. Pubertal development was recorded by the pediatrician according to Tanner [27].

### 2.4. Mixed Meal Tolerance Test

The children were asked to consume a daily amount of at least 150 g carbohydrates three days prior to the mixed meal tolerance test and to continue their normal daily physical activities. The day before the test the subjects were asked not to consume any food or drinks after 10 pm, with the exception of tap water. On the morning of the mixed meal tolerance test, the fasting state was verbally confirmed by the participant and a parent. A catheter was placed in an antecubital vein for blood sampling. Fasting blood samples were taken twice with a 15-minute interval (*t* = −15 and *t* = 0). After the second fasting blood sample was taken, the participants received a mixed meal bolus of 200 mL (Nutridrink Yoghurt Style, Nutricia, Zoetermeer, The Netherlands). The mixed meal bolus consisted of 49% carbohydrates, 35% lipids, and 16% proteins, with a total caloric content of 300 kcal. After the consumption of the mixed meal bolus, blood samples were taken two more times with a 15-minute interval (*t* = 15 and *t* = 30) and four times with 30-minute intervals (*t* = 60, *t* = 90, *t* = 120, and *t* = 150). Blood samples were analyzed for total ghrelin, PYY, GLP-1, glucose, and insulin.

### 2.5. Laboratory Analysis

Tubes for blood collection of GLP-1, PYY, and ghrelin and the inhibitor dipeptidyl peptidase IV (DPP-IV) (Linco Research, St. Charles, MO, USA) were placed on ice. Immediately after blood sampling, DPP-IV was added to tubes for measurement of GLP-1 and PYY to prevent degradation of these hormones. Blood samples for GLP-1, PYY, and ghrelin were centrifuged within one hour after sampling and stored at −80°C until analyzed. Baseline and T2 samples were analyzed in the same batches for all three hormones. Serum GLP-1 concentrations were measured by a highly specific enzyme-linked immunosorbent assay (human active GLP-1 ELISA Kit EGLP-35K, Linco Research, St. Charles, MO, USA; intra-assay coefficient of variation 8 ± 4.8%; interassay coefficient of variation 7.4 ± 1.1%; sensitivity 2 pM/L). PYY (3–36) concentration was determined by human radioimmunoassay (RIA) (PYY-67HK RIA Kit A 0.056, LincoResearch, St. Charles, MO, USA; intra-assay coefficient of variation 5.5–8.5%; interassay coefficient of variation 2.9–9.4%; accuracy 86.9 ± 5.2%). Total serum ghrelin was determined by human RIA as well (Ghrelin Total RIA GHRT-89HK Kit A 0.056 MBQ, Linco Research, St. Charles, MO, USA; intra-assay coefficient of variation 0.9–1.3%; interassay coefficient of variation 6.2–7.8%; sensitivity 30 pg/mL). 

Glucose was analyzed from lithium heparine plasma by the glucose-oxydase method with the Unicel D × C 800 (Beckman Coulter, Woerden, the Netherlands). Plasma insulin concentration was measured by RIA (DSL-1600, Beckman Coulter, Woerden, the Netherlands; intra-assay coefficient of variation 4.5–8.3%; interassay coefficient of variation 4.7–12.2%; sensitivity 1.3 *μ*U/mL). An index for insulin resistance was calculated according to the Homeostasis Assessment Model for insulin resistance (HOMA-IR) formula: (fasting insulin (*μ*U/mL) × fasting glucose (mmol/L))/22.5 [28].

### 2.6. Statistical Analysis

The analysis was performed using the Statistical Package for Social Science SPSS, version 17.0 for Windows (SPSS Inc., Chicago, IL, USA), and the level of significance was set at *P* < .05. Data were checked for normality before analysis, using descriptive statistics for skewness, kurtosis, and Shapiro-Wilk test. The outcome variables for insulin were transformed to the natural logarithm. Data were expressed as mean ± standard deviation (continuous variables) and as count and percentage (categorical variables) unless otherwise defined. Values for the area under the curve (AUC) of 165 min (from *t* = −15 to *t* = 150 min) were calculated according to the trapezoid rule.

Paired *t*-tests were used to explore difference of the outcome variables after treatment, in total 16 paired *t*-tests were performed. A separate ANCOVA analysis was performed to determine if the change in AUC of the gut hormones over time (T2 versus T1) was effected by gender, age, pubertal stage, or change in BMI-SDS. In the ANCOVA analysis, the changes in AUC of the gut hormones were used as dependent variables, time (T2 versus T1) and gender as fixed variables, and age, pubertal stage, and change in BMI-SDS as covariates.

## 3. Results

### 3.1. Subjects

Forty subjects (18M/22F; age: 13.3 ± 2.0 years) were enrolled in the study, and 36 children completed both study occasions. One subject withdrew before all baseline measurements were completed, one discontinued treatment and two did not show up at T2 for personal reasons. No significant difference in baseline BMI-SDS or age was observed between completers (4.2 ± 0.7 SDS; 13.3 ± 1.8 years) and noncompleters (4.3 ± 0.3; 13.3 ± 3.1 years). Results are presented for the 36 children completing both study occasions (18M/18F).

### 3.2. Anthropometry and Metabolic Parameters

Changes between both study occasions in anthropometry and metabolic parameters are shown in Table 2. A significant reduction of BMI-SDS was found after 3 months of multidisciplinary lifestyle treatment. 

**Figure 1:**
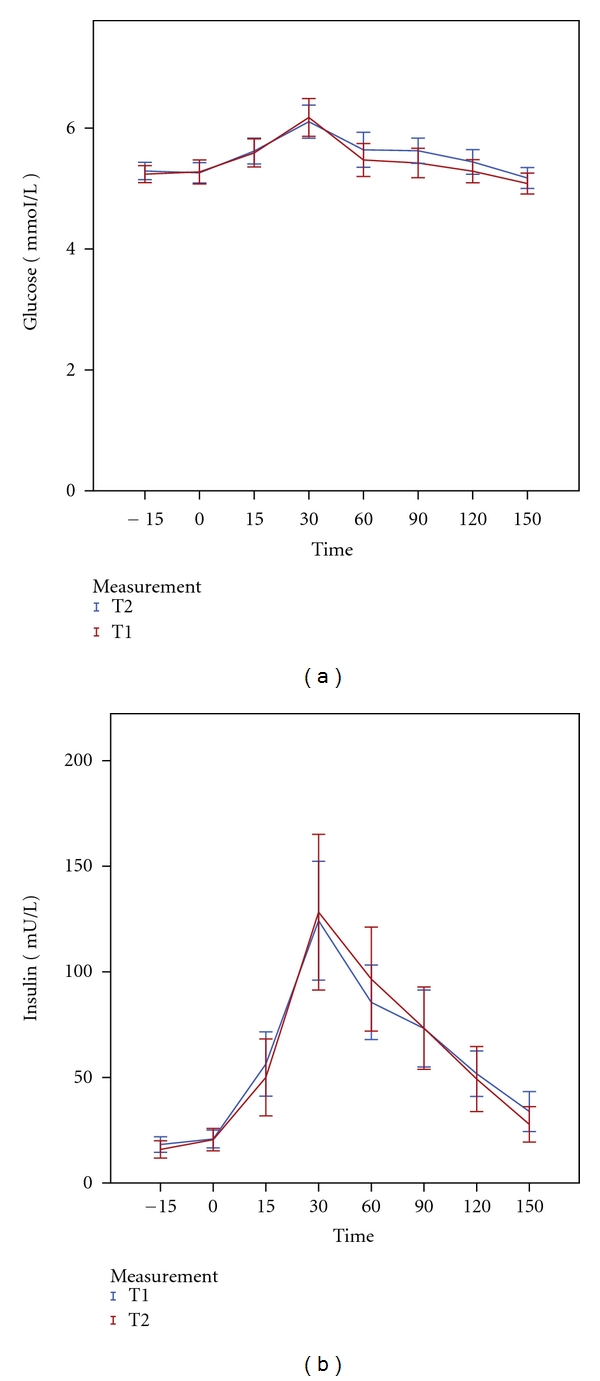
Mean plasma levels (±2SE) of Glucose (a) and Insulin (b) in response to a mixed meal at baseline (T1) versus after intervention (T2).

Fasting plasma glucose and insulin concentrations (and consequently HOMA-IR) were similar at baseline (T1) and after treatment (T2). The AUC of plasma glucose and insulin concentrations in response to the meal was also not affected by intervention (Figures 1(a) and 1(b)).

### 3.3. Gut Hormones

**Figure 2:**
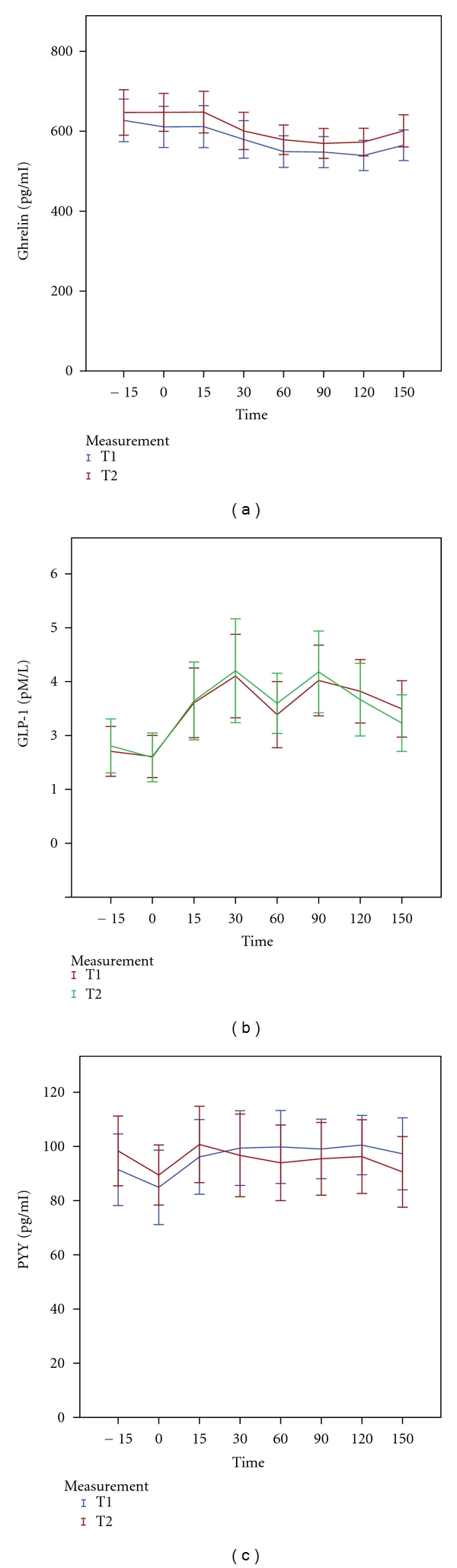
Mean plasma levels (±2SE) of Ghrelin (a), GLP-1 (b), and PYY (c) in response to a mixed meal at baseline (T1) versus after intervention (T2).

The ghrelin AUC in response to the meal was slightly, but significantly increased after treatment. Fasting levels were not significantly affected (Figure 2(a)). The treatment did not affect either fasting or postprandial plasma GLP-1 and PYY concentrations (Figures 2(b) and 2(c)). 

The ANCOVA analysis for change of circulating ghrelin and PYY levels between the two study occasions showed that the change in BMI-SDS during the study period was a significant covariate (*P* < .01 and *P* = .01, resp.). This finding suggests that weight loss affects the plasma PYY concentration. Gender, age, and pubertal stage were no significant covariates.

## 4. Discussion

A 3-month multidisciplinary lifestyle intervention in obese children reduces adiposity (BMI-SDS) and elevates plasma ghrelin levels in response to a meal. In contrast, it does not affect circulating PYY and GLP-1 concentrations in fasting condition nor in response to food intake. Notably, changes of ghrelin and PYY AUC were significantly influenced by the change of BMI-SDS, which suggests that the production of these gut peptides is sensitive to body weight (change). We speculate that the impact of our intervention on BMI was too modest to reach statistical significance for the difference between average levels. 

Various studies have shown an increase of fasting plasma ghrelin levels after weight loss in obese children [8, 10, 13]. However, the amount of weight lost was substantially greater in these studies than in ours. Other studies in which weight loss was more modest did not observe significant changes of fasting circulating ghrelin levels either [5, 14]. Interestingly, the change of plasma ghrelin concentrations was significantly and inversely associated with the change of BMI-SDS, which confirms that body weight (or caloric restriction) does influence ghrelin levels. This suggests that the effect of our intervention on weight reduction was too small to affect average fasting circulating ghrelin. It has been suggested that weight loss achieved by dietary or exercise treatment initiates compensatory changes in appetite and energy expenditure that hinders maintenance of the reduced body weight [4, 5]. Ghrelin release increases before a meal to initiate food intake [4, 29]. Thus, it is conceivable that higher (fasting) ghrelin concentrations in response to weight loss set off a greater hunger signal, which obviously renders the treatment goal more difficult to reach. 

It is unclear why serum ghrelin levels are low in obese children. Perhaps overfeeding is involved (feeding dampens ghrelin release) and the metabolic changes associated with obesity, such as insulin resistance [4]. Indeed, a previous study revealed that the change of HOMA-IR after weight loss correlates inversely with the change in plasma ghrelin in obese children, suggesting that these changes are mechanistically linked [5, 8].

GLP-1 and PYY are gut peptides produced in response to food intake. Both peptides provide a satiety signal to the brain to terminate eating [4]. Fasting plasma concentrations of either peptide were reported to be normal [9, 15–18] or reduced [19, 20] in obese children, whereas postprandial levels are consistently lower [15, 20, 21]. We show that modest weight reduction does not have an impact on fasting or postprandial GLP-1 and PYY levels in obese children. This is in apparent contrast to the results of the very few previous studies evaluating the effect of weight loss on plasma gut hormone levels in children. Substantial weight loss (≥0.5 BMI-SDS) appears to be accompanied by an increase in fasting PYY [19] and (surprisingly) a decrease in (fasting) GLP-1 [15, 18]. 

It is important to emphasize that our multidisciplinary lifestyle treatment resulted in a statistically significant but only modest weight reduction. It is therefore conceivable that a more substantial average weight reduction of ≥0.5 BMI-SDS would have had more explicit effects on (average) gut hormone concentrations. This postulate is supported by other studies, but also by our own data showing that the amount of weight loss is correlated with counterregulatory changes of plasma ghrelin and PYY concentrations, which can contribute to the difficulties that patients encounter when they try to lose weight. Thus, GLP-1 analogues or dipeptidyl-peptidase IV inhibitors may help obese children to lose weight in response to lifestyle measures. 

In conclusion, only a few studies so far have evaluated the effect of weight reduction on (postprandial) PYY, GLP-1, or ghrelin concentrations in children with obesity. We show that modest weight reduction by lifestyle intervention slightly elevates ghrelin levels, whereas it does not affect PYY or GLP-1 concentrations.

##  Conflict of Interests

The study was partly funded by an unrestricted educational grant by Pfizer and an unrestricted educational grant by a nonprofit foundation (de Stichting Vrienden van het JKZ). The sponsors had no role in the study design, data collection and analysis, nor the content of the paper. The corresponding author has full access to all data in the study. All authors declare no conflict of interests. This research was carried out at the Department of Pediatrics, Juliana Children's Hospital, The Hague, The Netherlands.

## Figures and Tables

**Table 1:** Characteristics of the family-based multidisciplinary lifestyle intervention.

Components	Description
Screening phase/individual counseling	During the screening phase, children and their parents were interviewed at two separate occasions and all individually by the multidisciplinary team consisting of a dietitian, a child physiotherapist, a child psychologist, and a social worker. Throughout those two separate meetings, an individual advice was given by the multidisciplinary team, based on the personal situation of each participating family.

Individual nutritional advice	A 3-day dietary recall (1 weekend day included) was used by the dietitian to get more insight in dietary habits of the children. Information was provided about nutrition and healthy eating behavior according to the traffic light nutritional list [25]. The traffic light nutritional list identifies several main food groups (fruits, vegetables, grains, milk and other dairy products, meat, fish, and others). Foods within each group were color-coded so as to reflect the caloric density per average serving and Dutch standards for healthy nutrition. The colors are green for “go”, orange for “approach with caution”, and red for “stop”. The children and parents were involved in planning their own daily diet for themselves according to the traffic light nutritional list.

Individual physical activity counseling	To obtain insight in the child's general physical activity behavior during the week, a physical activity questionnaire was filled out by a child physiotherapist. Children were asked about how they traveled to school (by foot, by bicycle, by public transportation, or by car), physical fitness classes at school, spire time sport activities, and daily computer and TV use as well as the duration spend at all these activities. The information from this questionnaire was used for advice on how to increase and optimize physical activity during everyday life, such as walking to nearby destinations and reducing sedentary activities (computer and TV use).

Individual psychological counseling	By means of motivational interviewing, the child psychologist helped the children to adapting to a new lifestyle in order to reduce body weight. Before the child started with the group sessions, individual treatment goals (reduction of 10% of body weight during 3 months of group sessions) were written down in a contract to avoid disappointment.

Children's group meetings	Most children with obesity have negative experiences with group activities. For example, they are often not included in social events or chosen last by peers during sport activities. Therefore, during the first session much time is spent in getting acquainted with each other. A good group bond is important for the effect of the treatment because peer support can be very helpful in the treatment of obese children. The main educational focus of the first two meetings is on nutritional information of a healthy eating pattern and the balance between energy intake and energy expenditure. During the subsequent meetings, emphasis was put in self-control techniques to cope with difficult situations (e.g., birthday parties, holidays, lunch breaks at school, being at home alone). Problem solving alternatives are debated (e.g., to avoid a situation, doing something else, participate in a situation and eat less, or participate followed by extra exercise afterwards). Other psychoeducational topics reviewed were self-reward (when coping well with a difficult situation) and self-regulation situations (making a plan how to integrate healthy behavior in daily living). Stimulus control was also one of the psychoeducational topics (remove unhealthy stimuli at home, encourage healthy behavior, eat at the dinner table, reduction of environmental stimuli linked to eating). Topics of the last two meetings were self-image (focus on positive things about themselves) and coping strategies on dealing with teasing.

Parent group meetings	Topics discussed during the parent meetings included the necessity to change their own lifestyle as well, information on healthy nutrition (product information, quantities, eating moments, eating locations), and how to help their children. Parents received advice on parenting styles (boundaries setting with regard to eating behavior, giving positive feedback). During the last meeting, a therapist discusses the role of all other family members with regard to the treatment in the family (e.g., are other family members supportive, how do they cope with the lifestyle changes).

Follow-up meetings	In order to maintain the newly learned behavior, refresh follow-up meetings were given during the first two years (2-3 meetings/year). The child psychologist and the social worker organize this follow-up meetings. The topics repeated were problem solving techniques and relapse prevention techniques.

**Table 2:** Clinical and physiological parameters of the subjects with complete data (*N* = 36).

Variables	Baseline (T1)	After (T2)	*P* Value*
BMI-SDS	4.2 ± 0.7	4.0 ± 0.9	.003
HOMA-IR	4.2 ± 2.5	4.1 ± 3.0	NS
Glucose (fasting) (mmol/L)	5.2 ± 0.4	5.3 ± 0.4	NS
Glucose AUC (165 min × mmol/L)	905 ± 67	896 ± 86	NS
Insulin (fasting) (mU/L)	18.0 ± 10.7	17.4 ± 12.5	NS
Insulin AUC (165 min × U/L)^†^	10.3 ± 6.1	11.0 ± 8.0	NS
Ghrelin (fasting) (pg/mL)	612 ± 143	641 ± 144	NS
Ghrelin AUC (165 min × pg/L)^†^	92.3 ± 18.3	97.9 ± 18.2	.006
PYY (fasting) (pg/mL)	90.2 ± 34.6	95.5 ± 32.8	NS
PYY AUC (165 min × pg/L)	16.3 ± 4.6	15.3 ± 5.0	NS
GLP-1 (fasting) (pM/L)	2.6 ± 0.9	2.5 ± 0.9	NS
GLP-1 AUC (165 min × pM/L)	567 ± 192	552 ± 197	NS

*P value T2 versus T1 (paired *t*-test).
